# Measured oxygen levels in Norwegian waters and implications for future offshore Atlantic salmon aquaculture

**DOI:** 10.1038/s41598-025-12697-x

**Published:** 2025-08-11

**Authors:** Øystein Skagseth, Frode Oppedal, Henrik Søiland, Malthe Hvas

**Affiliations:** Norwegian Institute of Marine Research, Bergen, Norway

**Keywords:** Fish welfare, Oxygen, Hypoxia, Normoxia, Ocean surveys, Seasonal variability, Norwegian continental shelf, Environmental sciences, Ocean sciences

## Abstract

Offshore salmon farms are being planned on the Norwegian Shelf. However, there are concerns of low oxygen levels within sea cages during periods of low water exchange. Hypoxia will compromise fish welfare and production performance, and it is therefore important to characterize oxygen dynamics at new offshore aquaculture sites. Here, we investigated oxygen variability in Norwegian waters by utilizing data from repeated sections and fisheries surveys collected between 2010 and 2023. In total more than 5000 profiles were analysed. Hypoxia was regularly observed in the inshore and fjord areas. However, no evidence for severe hypoxic conditions were seen on the shelf regions despite nutrients input being rather high. This lack of hypoxia at depth suggests that the cross-shelf exchange supplying oceanic water dominates the oxygen balance at the shelf relative to biological consumption. Meanwhile, seasonal cycles in oxygen, apparent oxygen utilization, temperature, and stratification were observed. Notably, oxygen saturations of 70–80% occasionally occurred offshore below the mixed layer during summer and fall. Such moderate hypoxia represents the initial conditions to be considered when planning cage sizes and allowable biomass. Since oxygen will not always be fully saturated, challenges with hypoxia within sea cages will likely prevail when moving offshore.

## Introduction

In Norway, Atlantic salmon (*Salmo salar*) aquaculture has traditionally used sea cages located near shore and within sheltered fjord environments^1,2^. More than 1,500,000 ton of salmon and about 90,000 ton rainbow trout (*Oncorhynchus mykiss*) are produced and traded yearly^3^. However, owing to negative environmental impacts, which in particular includes the imposed infestation pressure of sea lice on wild salmonid populations^4^, the sustainable limit for allowable farm sites has been reached. Further growth of the Norwegian salmon industry is therefore now dependent on developing alternative methods of fish farming^5,6^.

A potential solution for sustainable growth is to develop offshore Atlantic salmon aquaculture^7,8^. Offshore aquaculture is defined as farm sites located more than 2 km from the coast, in waters deeper than 50 m, and with occasional exposure to powerful waves and strong water currents^9^. Going offshore should offer several noteworthy advantages such as higher water quality owing to increased water flow through the cage structures, which removes waste products and provides a homogenous sea cage environment with excellent oxygen conditions^10,11^. Furthermore, parasite and pathogen transmissions between sites will presumably be reduced owing to greater hydrographic distances, while fewer conflicts of interest with other stakeholders are expected since offshore farms will be located out of sight and further away from populated areas^7,12^. Meanwhile, going offshore poses many new challenges and requirements regarding technology and infrastructure, as well as an increased complexity in all operational tasks and work routines^13–16^.

Fish welfare considerations are crucial when developing new aquaculture practices. A central question for the feasibility of offshore aquaculture has here been whether the fish can thrive at sea in more extreme environments. So far, the primary fish welfare concern has been the exposure to powerful waves and strong water currents, and much effort has therefore been made to define biological limits of farmed Atlantic salmon to such conditions^17–20^. Considering surveyed water current conditions at candidate offshore areas together with the fact that Atlantic salmon are powerful sustained swimmers, it is presently expected that the fish will be able to thrive in these exposed environments^11,21^.

Another potential fish welfare challenge exists that paradoxically is not caused by stormy weather conditions, but instead by the occurrence of prolonged calm periods with little or no water currents, particularly during summer. Low current conditions will limit water exchange rates through the sea cage and thereby increase the risk of environmental hypoxia (low oxygen levels)^22–28^. Periods of low current conditions may also increase the rate of biofouling on nets which can cause additional challenges with inadequate water exchange through sea cages^29^. Hypoxia risks may be amplified further at offshore sites because the industry is planning to use larger cage designs that holds substantially larger biomasses than at traditional farm sites^30^, which will impose inherent higher requirements for adequate water exchange rates^23^. In two recent reviews it is clearly stated that future studies need to address how the water flow changes through biomasses of 1000–10,000 tons of fish in a cage and the consequences on oxygen levels^31,32^. Hence, both periods of too fast and too slow water currents may become a fish welfare issue at offshore aquaculture sites.

**Fig. 1:**
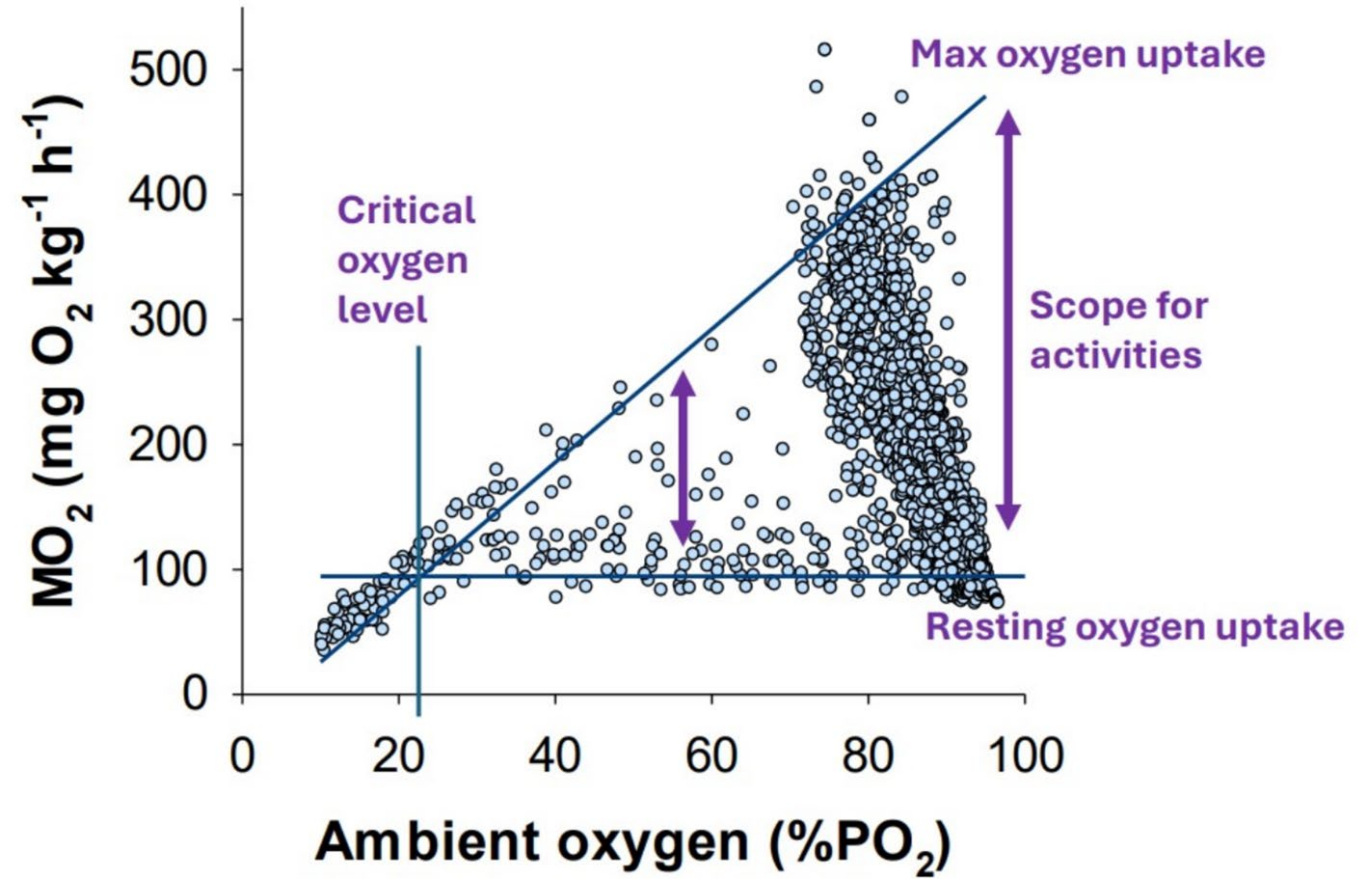
Oxygen uptake rate (MO_2_) dynamics in Atlantic salmon at different ambient oxygen levels. The maximum oxygen uptake rate decreases steadily with declining ambient oxygen levels while resting oxygen uptake rates remains the same until a critical point, and below this point survival is not possible. The scope for activities marks the difference between maximum and resting oxygen uptake rates and gradually declines towards zero at the critical oxygen level. Key activities (e.g., feeding, swimming, immune responses) all require a portion of this scope, and these functions therefore become more and more restricted as water becomes more hypoxic. Data shown was measured with respirometry using Atlantic salmon of ≈ 4 kg at 9 °C in full strength seawater (modified from Hvas et al., *in review*).

Dissolved oxygen is one of the most important water quality parameters in aquaculture as insufficient levels will have detrimental effects on the fish. Tolerance limits to environmental hypoxia will depend on the type of activity together with the interactive effect of water temperature. As such, hypoxia tolerance in fish generally decreases with increasing temperature because oxygen requirements of the fish increase owing to elevated metabolic rates while the water solubility of oxygen decreases. To sustain key activities such as feeding, digestion, and swimming, sufficient oxygen levels are required, and with increasing hypoxia these activities become progressively more restricted. In severe hypoxia, the fish will become lethargic as it only is able to support its most basal metabolic needs to avoid suffocation. Below a certain threshold, even basal needs cannot be sustained aerobically, and survival becomes severely time limited (Fig. 1).

In the case of farmed Atlantic salmon, various tolerance limits to environmental hypoxia are documented in experimental studies. These include interactions with temperature on feed intake, swimming capacities across size classes, and the critical limits for acute survival^33–36^. Reported oxygen threshold values can subsequently be used to evaluate sea cage environments. For instance, Atlantic salmon at traditional Norwegian sites have been found to occasionally be subjected to appetite limiting conditions owing to insufficient oxygen levels in sea cages^23,37,38^. In warmer regions of Atlantic salmon aquaculture, more extreme hypoxia reports exist that periodically would make the fish voluntarily anorexic^39,40^. To evaluate the risks of detrimental hypoxia levels in offshore sea cages we already have a good data fundament of welfare requirements and consumption rates in farmed Atlantic salmon. The remaining question therefore pertains to oxygen conditions of the shelf water, i.e. the water representing the initial condition for the further oxygen consumption by the salmon within the cages.

The flux of oxygen across the air-ocean interface depends on the diffusion across a thin laminar layer that critically depend on the wind velocity^41^. For a large scale mean mixed layer depth of 80 m, the equilibrium time scale is about 1 month^42^. However, in the Norwegian Coastal Waters the mixed layer depth is shallower^43^, and thus the equilibrium time scale becomes shorter. Generally, the ocean surface layer is within about 2% of full oxygen saturation^42^, except for regions of deep-water formation, coastal upwelling, anthropogenic changes, and sea-ice^44–46^. Depletion of oxygen can be inferred as a measure of respiration. Surface temperature is a key driver of change since it both affects the solubility of oxygen, but also alters the stratification, and thus alters the transport from where oxygen is reset by air-ocean exchange and produced by photosynthesis, to the deeper waters where it is consumed by heterotrophic processes^47^.

In well mixed oceanic shelves, oxygen levels are not considered a problem for fish species or the marine ecosystem generally. This is especially true in relative shallow seas such as the southern North Sea^48,49^. Here strong tidal currents provide mixing energy to cause weak stratification, and conditions for a downward flux of surface water saturated in oxygen ^50^. Further north in the North Sea and on the Norwegian shelf, bottom depths are larger, and tidal currents are less vigorous which reduces the potential for a downward oxygen flux. During winter the frequency of storms is relatively high and associated wind mixing will contribute to increased oxygen levels at depth. However, during summer calmer wind conditions and surface warming leads to less oceanic uptake of oxygen^51^.

For fjord-like areas and protected coastal basins calmer waters and vertical stratifications in temperature and salinity create density gradients that may limit oxygen flux downwards^52,53^. Within these waters most of the salmonid aquaculture production occurs in Norway, and at many sites the fish cages face lowered oxygen levels already with the incoming water. The extent and consequence of this are presently vaguely understood and needs to be addressed further.

The objective of this study was to provide a comprehensive report on the oxygen conditions in representative offshore Norwegian waters throughout different seasons, and to compare this data with traditional fjord and coastal sites. Specifically, we aimed to assess offshore oxygen conditions from the perspective of Atlantic salmon aquaculture, focusing on fish welfare and overall production performance metrics based on known hypoxia thresholds for this species. We anticipated that oxygen conditions would be better offshore compared to inshore fjord sites. However, we suspected that deeper waters in late summer and early autumn might exhibit some degree of environmental hypoxia. A key question was therefore whether these conditions could be sufficiently severe to negatively impact aquaculture production metrics and fish welfare.

## Data and methods

### Data collection

**Fig. 2:**
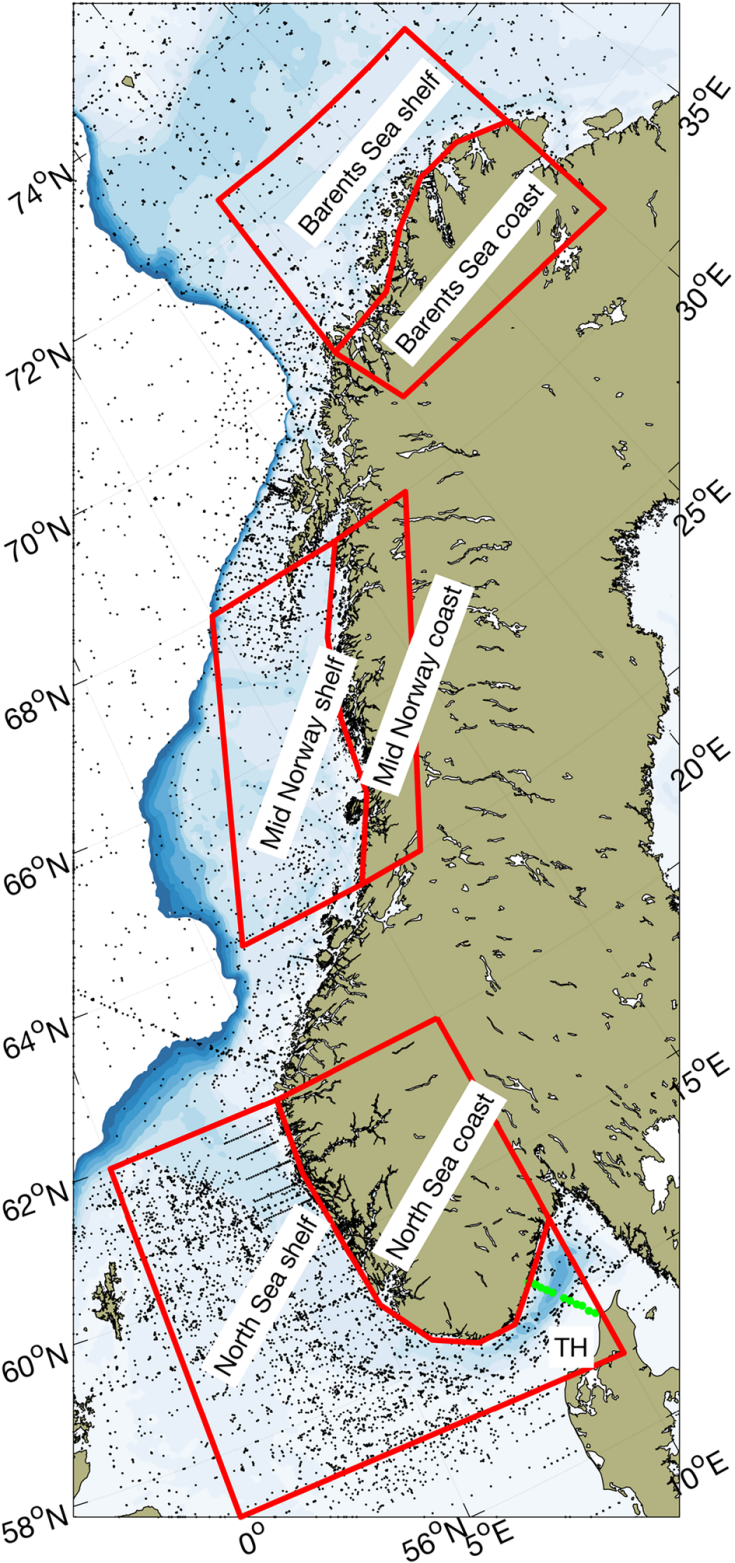
Map of the study area. Bottom topography in color and black points denote CTD-O (Conductvity, Temperature, Depth, Oxygen) profiles obtained from ships. The Torungen-Hirtshals (TH) section is shown as green points. The CTD-O data are sorted into shelf and coastal for the three regions North Sea, Mid Norway, and Barents Sea.

The Norwegian Institute of Marine Research samples a range of vertical environmental data every year on regular and random positions at the shelf and coastal regions of Norway during cruises with its own vessels and with commercial fishing vessels. For this descriptive study of environmental oxygen levels, we used measurements taken in the period 2010–2023. These measurements were then divided between the Norwegian shelf and coastal waters within three regions: North Sea, Mid Norway and Barents Sea (Fig. 2).

**Fig. 3:**
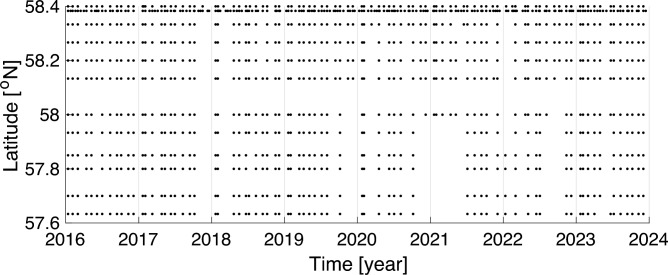
Coverage from the Torungen-Hirtshals section. Data consists of 12 hydrographic stations where water are sampled at depths of [0, 5, 10, 20, 30, 50, 75, 100, (125), (150), 200, 300, 400, 500, 600] meter at monthly intervals.

Oxygen conditions were investigated using a combination of water samples with titration analysis of oxygen and direct measurement of oxygen with a probe. First, we used data from the Torungen-Hirtshals hydrographic section (see Fig. 2) operated at nominally monthly intervals (Fig. 3). Water samples for oxygen analysis were here taken at standard depths [0 5 10 20 30 50 75 100 (125) (150) 200 300 400 500 600] meter but limited to 12 bottles per profile. Each bottle sample was analyzed for oxygen using Winkler titration that are accurate to about 0.1%^54,55^. A Seabird 911 CTD (Conductivity, Temperature, Depth) probe was mounted on the same water sampler rig providing simultaneous temperature, salinity and pressure data. Measurements from 1066 vertical profiles obtained between 2016 and 2023 from the Torungen-Hirtshals section were used (Fig. 3).

**Fig. 4:**
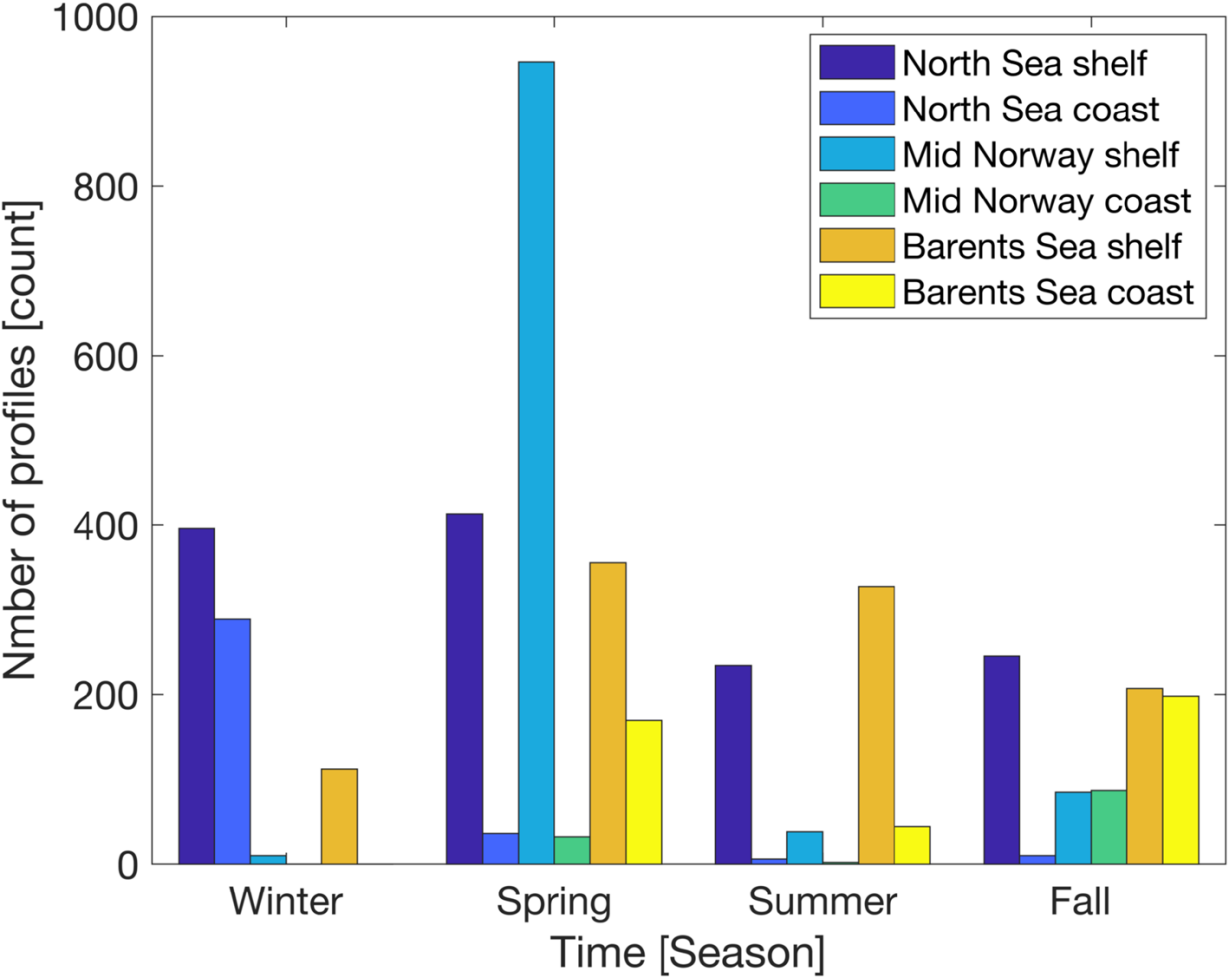
Number of ship profiles (CTD-O) taken for measurements of oxygen. The counts are given for winter (December, January, February), spring (March, April, May), summer (June, July, August) and fall seasons (September, October, November), and divided into shelf and coastal regions of the North Sea, Mid Norway and Barents Sea according to the areas shown in Fig. 2.

To obtain a broader spatial variability representing the entire Norwegian coast and shelf we utilized data sampled during fisheries—and ecosystem surveys as well as from repeated hydrographic sections. To provide robust estimates of oxygen we grouped the profiles according to the regions shown in Fig. 2. In total, 4241 vertical profiles sampled between 2010 and 2023 were used for this analysis. The profiles were measured with a Seabird 911 CTD system with an SBE43 oxygen optode. A typical profile was taken by reducing vessel speed to zero and lowering the monitoring rig from the surface to increasing predetermined depths, depending on the need for data at each specific position. The number of profiles were not evenly distributed across seasons and the geographic areas surveyed, and some areas therefore have less data (Fig. 4). For both the North Sea and Barents Sea shelf the coverage is generally good in all seasons, while for the Mid Norway shelf the sampling is very high in spring but rather limited during the other seasons. The coverage at coastal regions is generally less frequent and also vary between seasons.

### Optode sample principle, drift, and correction of oxygen data

The SBE43 oxygen optode probe determines dissolved oxygen concentration by counting the number of oxygen molecules per second (flux) that diffuse through the membrane from the ocean environment to the working electrode. However, this sensor electrolyte changes continuously as oxygen is measured, resulting in a drift over time^56^. Despite annual service of the optode that includes calibration, this drift will appear as a constant bias for each profile in the oxygen data obtained by the optode.

The method to deal with the drift in the oxygen optode is based on the relative rapid rate of gas exchange of oxygen at the air ocean interface^57^. Broecker and Peng^42^ give an equilibrium time scale of one month assuming a typical mixed layer depth (MLD) of 60 m. The MLD is shallower in Norwegian shelf and coastal waters and are typically less than 30 m. Since the equilibrium time scale is proportional to the MLD^42^, the saturation time scale of oxygen in our study would be in the order two weeks. Based on this we correct the oxygen bias by assuming 100% oxygen saturation at the surface, a function of temperature and salinity, according to

1 $${O{\prime}(z)}_{optode}={O(z)}_{optode}+{[O\left(z=0\right)}_{saturation}-{O\left(z=0\right)}_{optode }]$$

where O’(z) optode is the corrected profile, and on the right hand side the 1st term is the measured profile, and the 2^nd^ term is the difference between the calculated oxygen saturation^51^ and measured oxygen in the surface layer. We acknowledge that this approximation relies on a few simplifications. First, the air-sea gas exchange at the surface is not constant but depend on the thickness of the laminar layer that further is affected by the turbulent structure of the water beneath that largely is dependent on the wind speed^41^, and as noted above on the MLD^42^. Second, oxygen is produced by photosynthesis and consumed by respiration. However, the high monthly sampling rate at the Torungen-Hirtshals section with Winkler oxygen titration allow us to accurately assess the mean monthly saturation and biological contribution, the so-called Apparent Oxygen Utilization (AOU)

2 $${O}_{AOU\_mon}=\frac{1}{n}\sum_{j=1}^{n}\left({O\left(z\right)}_{Winkler}-{O\left(z\right)}_{saturation}\right)$$

where n is the number of profiles for a given month, and the right hand side represent the difference between the measured Winkler oxygen data and the calculated 100% oxygen saturation based on the measured temperature and salinity^51^.

### Data analyses and presentation

The analysis and presentation of data is broadly structured in the following manner: First, we utilized the oxygen measurements from the Torungen-Hirtshals section obtained by Winkler titration. This analysis provided the basis to evaluate the assumptions used to correct oxygen profiles obtained during regular CTD-O casts, and wherefrom we resolved the spatio-temporal variability in Norwegian shelf and coastal regions. To conform with the unevenly distributed numbers of CTD-O casts (Fig. 4), we categorized them into shelf or coast/fjord for each of the three geographical regions assessed (North Sea, Mid-Norway and Barents Sea) (Fig. 2). Since aquaculture cages are not expected to be located deeper than 100 m, we only considered the upper 150 m of the surveyed profiles.

To resolve the variability in oxygen concentration, including the biological contribution, we utilized the oxygen measurements analyzed with the Winkler method from the Torungen-Hirtshals section (Fig. 3). To achieve a robust estimate of the annual variability of the oxygen concentration, we calculated the across station depth-mean values of oxygen. Based on the same dataset, we resolved the effect of biological production, referred to as apparent oxygen utilization (AOU), by presenting the difference between the observed oxygen and the calculated oxygen concentration at 100% saturation as a function of temperature and salinity. To further detail the oxygen variability in the surface water (< 5 m), we present a scatter plot of the individual samples and monthly sorted standard deviation of measured concentration in oxygen (mg l^-1^) with its corresponding calculated relative saturation of oxygen^51^.

All profiles taken with the optode probes are plotted and sorted by season defined as winter (December-February), spring (March–May), summer (June–August) and fall (September–November) for the three defined regions North Sea, Mid-Norway and Barents Sea within inshore and coastal areas. For each division of data we included the average, the ± 2 standard deviation and the lower 5-percentile**.** To further illustrate the fjord to shelf gradient in environmental oxygen we present profiles from the bottom of Førdefjorden (6°E) located in the North Sea region with extension to the outer shelf profiles at 2°E.

## Results

### Torungen—Hirtshals transect data

**Fig. 5:**
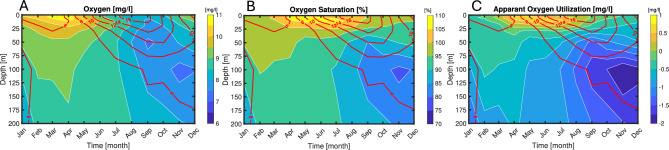
Mean month-depth oxygen variability in the Torungen-Hirtshals section. (**A**) Oxygen concentration (mg l^-1^), (**B**) Oxygen saturation (%) and (**C**) Apparent oxygen utilization (mg l^-1^) (AOU = O_2 measured_-O_2 saturation_) averaged per month (contour colour plot) over 2016–2023 and all positions of the Torungen-Hirtshals transects with temperature given as red isolines.

The oxygen measurements in the Torungen-Hirtshals section showed a pronounced seasonal cycle in oxygen concentrations over the focused upper 200 m (Fig. 5A). In the surface layer the maximum oxygen concentration coincides with the onset of the spring bloom around April with levels > 10.5 mg l^-1^. Coinciding with nutrient depletion in the surface layer in spring, the primary production and the associated oxygen maximum occurs sub-surface from May to August, but with decreasing values towards 9 mg l^-1^ as the temperature increases. Below the surface layer (50 to 200 m), the level of oxygen generally decreases from 9.5 to 8.5 mg l^-1^, and with absolute minimum values of about 7.5 mg l^-1^ around 100 m depth in late fall. In terms of relative oxygen saturation values exceeds 105% in the near surface layer during spring (Fig. 5B). During late summer and fall the oxygen saturation decrease with an absolute minimum below 80% at about 100 m depth in November. The temperature in the upper layer shows a seasonal pattern from 6 °C in winter and gradually increasing in spring and summer peaking at 16 °C in fall (August–September) following decrease in late fall into winter (Fig. 5). At depth there is a pronounced seasonally delayed warming due to downward mixing of the summer water, while deeper than 150 m the temperature shows minor seasonality.

The apparent oxygen utilization (AOU) shows a positive value in the surface layer from March through October due to net primary production (Fig. 5C). The absolute maximum, exceeding 0.75 mg l^-1^, is at the surface in April–May and then subsequently becomes subsurface and slightly delayed compared to the oxygen concentration. Then from September through March there is a minor negative AOU. Below the surface layer the AOU is always negative, and especially during fall with the absolute minimum in late fall at about 100 m depth of the order -2 mg l^-1^ at end of the year.

**Fig. 6:**
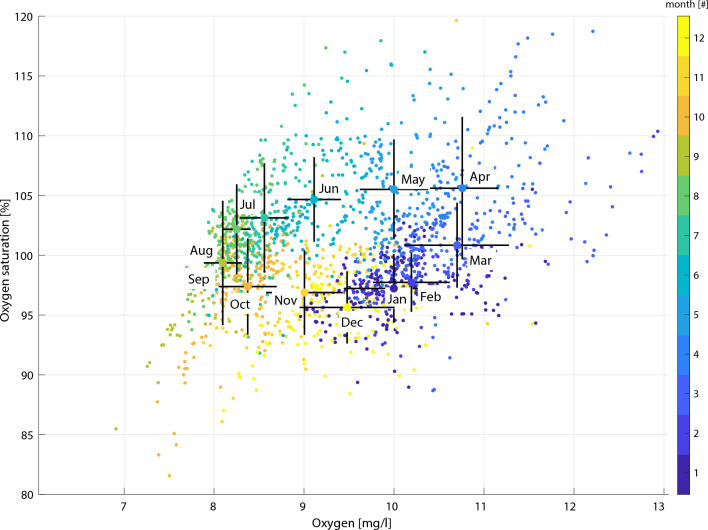
Near-surface oxygen measurements. Seasonal variability of oxygen measured (mg l^-1^) and oxygen saturation (%) in the surface layer (depth <  = 5m) for the Torungen-Hirtshals transect from January (dark blue) to December (yellow). Monthly averages displayed as colored points with standard deviation as crosses.

To further detail the seasonality of oxygen at the surface (< 5 m) we show the relation between measured concentrations and the saturation percentages sorted by month (Fig. 6). The monthly means of oxygen saturation ranged from 95 to 105%, with standard deviations of ± 5%, while oxygen concentrations ranged from 7.1 to 10.7 mg l^-1^ with standard deviations up to ± 1.3 mg l^-1^. There was a clear “annual wheel” in oxygen. That is, from September through February there is a moderate undersaturation (less than 5%) that peaks in December coinciding with increasing absolute oxygen concentrations due to decreasing surface temperatures which increase the solubility of oxygen. Starting in March there is a pronounced increase in oxygen saturations due to the onset of the spring bloom that peaks in April and May, and thereafter a gradual decrease in both saturation and absolute values through the summer as the temperature increases.

### Spatio-temporal variation in oxygen

**Fig. 7:**
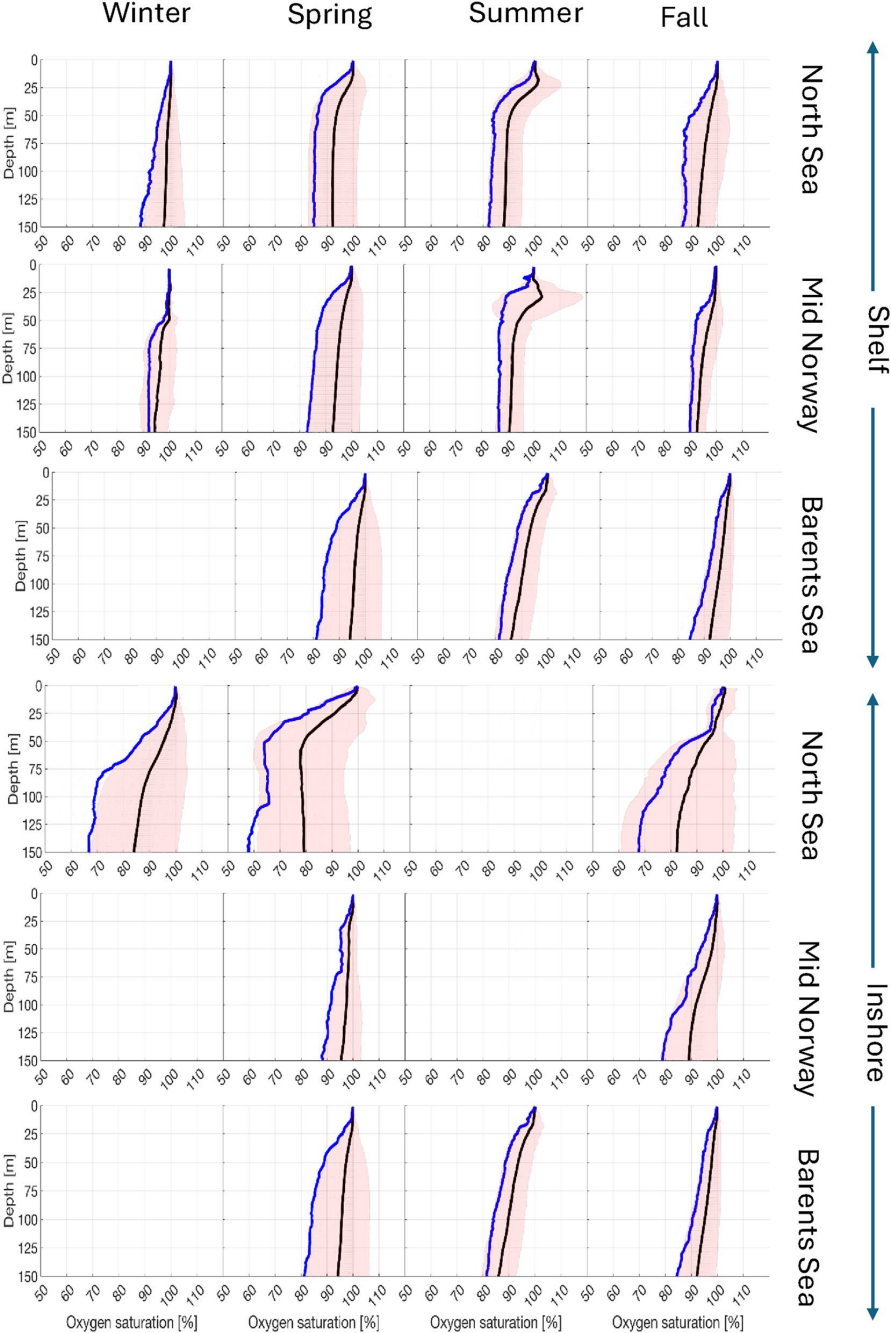
Regional oxygen saturation. Oxygen saturation versus depth profiles sorted into seasons, and shelf or inshore for North Sea, Mid-Norway and Barents Sea. Red area covers the mean ± 1 std, and blue lines are the lower 5%tile.

The oxygen profiles, expressed in relative oxygen saturation (%) with depth, sampled with the seabird CTD-O system are sorted seasonally and spatially into off- and inshore, and for the three regions; North Sea, Mid-Norway and Barents Sea (Fig. 7). From these data groupings, several patterns are clearly seen when considering the upper 100 m of waters where offshore salmon aquaculture is being planned.

We see that in offshore regions oxygen saturation is mostly normoxic, with values of 80–120%. Only a few exceptions are seen in Mid-Norway during spring and North Sea between spring and fall where some observations are down to about 75%. Generally, oxygen saturation is close to 100% in winter and both above and below from spring to fall with highest values closer to the surface during summer and lowest values at deeper depths > 50 m. Above 25–30 m depth, the oxygen is always > 95% saturation. For the offshore shelves the differences between the three regions are relatively similar. The 5%tile is overall above 80%, but with generally higher values northwards.

Large variations in oxygen are seen within the inshore regions, mainly for the North Sea, partly for the Barents Sea and only in fall of Mid-Norway. For the inshore region of the North Sea, conditions below 20–50 m depth are often seen to be moderately hypoxic (< 75%) to severely hypoxic (< 50%) from May, but worse in summer and fall. However, many profiles are normoxic (> 80%) at all depths throughout the year. Within Mid-Norway values < 80% is only seen in fall deeper than 100 m. However, it should be noted that profiles for Mid-Norway in winter and summer, and Barents Sea in winter are lacking. The inshore regions in Mid-Norway and Barents Sea have a 5%tile above 75% throughout all depths.

**Fig. 8:**
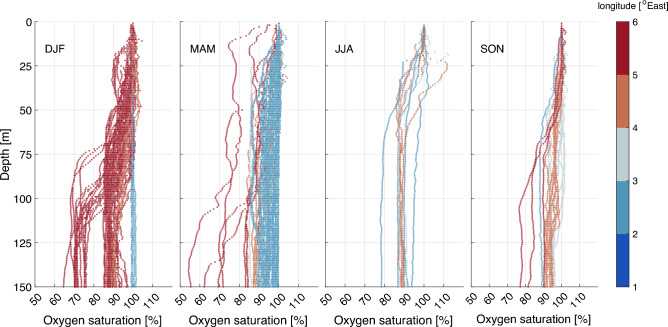
Inshore versus offshore oxygen saturation. Data are taken from the Førdefjorden section at about 61.5 ^o^N, from the shelf at 2 ^o^E to the inner fjord at 6 ^o^E. Panels show vertical profiles of oxygen saturation as a function of depth sorted by seasons (letters refer to months) with longitude in color bar.

The profiles from the inner parts of Førdefjorden (6°E) to the outer shelf area (2°E) illustrate some of the variations that occur from inshore and disappear towards the open waters (Fig. 8). Already in the coastal area (5°E), in winter and partly in spring, moderate hypoxia is seen from 50 m downwards with more severe hypoxia below 100 m depths. Normoxia is mainly seen in the upper 50 m, except some profiles that show values down towards 70% oxygen saturation in spring within the fjord. In the more westerly profiles (< 4.5°E), normoxia is seen with values > 80% and only minor stratifications of higher values close to the surface. It should be noted that more profiles within the fjord are taken during winter and most profiles at the shelf were sampled in spring.

## Discussion

Historical oxygen measurements in Norwegian offshore and inshore areas spanning 13 years were here compiled and presented. As anticipated, the data displayed seasonal and depth-related variations, with offshore areas primarily showing levels above 80% oxygen saturation while inshore fjord areas were more prone to severe hypoxia (> 60%). As such, when considering the ambitious plans of establishing offshore aquaculture sites on the Norwegian Shelf, environmental conditions were found to be mostly normoxic and should thus be adequate from a fish welfare and growth performance perspective most of the year. However, notably precautions were late summer and early autumn where offshore ambient oxygen levels may approach 80% saturation. This marks a low entry point prior to the fish consuming oxygen, especially if flow conditions and water exchange through the cage simultaneously are low. Seasonal limitation in biomass per sea cage may therefore be necessary to mitigate hypoxia risk and to safeguard fish welfare at future offshore aquaculture sites. Meanwhile, the documentation of both moderate and severe hypoxia within fjord areas also highlights the prevailing challenges with hypoxia events in present day salmon aquaculture in Norway. Considerations of seasonal and depth-related oceanic processes driving environmental oxygen variability could therefore inform better management of salmon aquaculture in both fjord, coastal, and offshore areas.

### Management of environmental oxygen levels at offshore aquaculture sites

The compilation of oxygen measurements within the Norwegian shelf and outer coastal waters represents 13 years and hundreds of stations sampled throughout all seasons. The levels of measured oxygen saturation were typically in the range from 80% to above 100% at the most relevant farming depths of 0–100 m. Generally, these observed oxygen levels at the Norwegian shelf are well above threshold levels that may cause challenges for farmed Atlantic salmon. For instance, the feed intake of 500 g Atlantic salmon declines when oxygen levels fall below a temperature dependent threshold. Specifically, for post-smolts at 19 °C oxygen levels above 75% saturation are required to maintain maximum feed intake, while this decreases approximately linearly with temperature down to 42% saturation at 7 °C^33^. Feed intake in larger Atlantic salmon of 2.3 kg was halved at oxygen saturation 70% compared to 100% within the relatively high temperature of 18 °C^58^. Moreover, feed intake was reduced by 90% at 60% compared to 100% oxygen saturation in 800 g Atlantic salmon^59^. At even lower oxygen levels, below the limiting oxygen saturation (LOS), the fish will become anaerobic and survival becomes acutely time limited. The LOS levels for post-smolt Atlantic salmon of 500 g range from 40 to 24% oxygen saturation for temperature between 19 and 7 °C^33^. The authors also state that these limits should be considered conservative, as thresholds may be higher for fish under commercial conditions where added daily challenges are seen within sea cages along with the additive impacts of parasites and pathogens^32,60,61^.

Oxygen levels offshore seem to be well above any welfare thresholds for Atlantic salmon. However, the consumption of oxygen by the fish themselves may create poor oxygen conditions within the cages if the biomass is higher than the exchange rate. Farmers will need to consider the constant oxygen consumption within a fully stocked sea cage where both the initial oxygen level of incoming water, the temperature, and water renewal rates by currents are key factors. Some recent works have estimated oxygen levels within cages by mathematical modelling of the oxygen budget^26–28,62^, and were able to temporarily match, but also mismatch the measured oxygen levels within and around the cages^26,62^. Oxygen levels below welfare thresholds have frequently been observed in present standard sized cages^22,37,38,63–65^. Furthermore, lower oxygen levels have been observed and modelled in large sized cages compared to smaller ones^27,28,35^. To provide realistic modelling of oxygen conditions in stocked sea cages, high quality data must be available for input. The present study can thereby contribute to more precise and dynamic oxygen modelling in stocked sea cages to mitigate hypoxia risks and inform biomass limitations. Important considerations here pertain to the fact that ambient oxygen levels at offshore sites will not always be fully saturated. For instance, during late summer and fall the shelf water that enters sea cages may approach 80%. This leaves little room for additional oxygen consumption by the fish being farmed to maintain adequate conditions for appetite and growth, depending on prevailing flow conditions, water exchange rates, and total biomass in the sea cage.

### Poorer oxygen conditions in inshore fjord areas

We observed a consistent decrease in environmental oxygen levels when moving from offshore waters out on the shelf towards the coast and further into the fjords. Similar patterns have been observed in Chilean Patagonian Fjords, which constitute the second largest global salmon farming area^52^. Mechanisms that contribute to more hypoxic conditions inshore includes land runoff that cause a fresh stable stratified surface layer together with generally weaker winds and waves limiting downward fluxes of oxygen. Furthermore, fjords in Norway generally have a sill, except for in the Barents Sea, limiting lateral exchange and renewal of the deeper layers. As such, in the example of Førdefjorden used in the present study, the lowest oxygen saturations (< 60%) occurred below 100 m coinciding with the depth of the sill. Renewal of water below the sill depends on vertical mixing in the basin and that the density of the inflowing coastal water is dense enough to replace the fjord water at depth^66^. In contrast to this, the offshore deeper water shows no sign of comparable low oxygen saturations.

### Ocean flows explain patterns in oxygen at the Norwegian Shelf

One may question the underlying cause of consistent normoxic conditions in the Norwegian Shelf considering the area being highly productive. The southern North Sea is unstratified due to shallow depths and strong mixing that effectively leads to a downward flux of recently ventilated water that is close to saturation. However, in the stratified area further north in the North Sea, below the pycnocline, lateral flux of oceanic origin may be more important. Considering the ratio between the volume of the North Sea at 5.4 × 10^4^ km^3^ to the rate of Atlantic inflow of about 2 Sverdrup (1 Sverdrup = 10^6^ m^3^ s^-1^^67^; give a mean residence time in the order of < 1 year. Downstream from the North Sea, the salinity of the Norwegian shelf waters increases with an order of 0.001 psu (practical salinity unit) km^-1^. When assuming that the volume flux of the Norwegian Coastal Current is constant, this requires an overall cross slope transport of order 1 m^2^ s^-1^ of Atlantic Water with O_2_ 300 micro mol kg^-1^. The exchange between shelf—and Atlantic Water is therefore a major source of oxygen renewal at depth on the shelf. This oceanic oxygen flux should still work and possibly become more important for maintaining high oxygen levels at depth under future warming with less oxygen uptake at the surface layer and presumed stronger stratification and reduced vertical mixing.

### Upwelling deep-water hypoxia

There are widespread concerns that an increasingly low oxygen oceanic layer will develop owing to global warming^46^. During upwelling conditions hypoxic water are observed to enter the continental shelf, for instance as seen on the US West coast^68^, in the southern part of the Canary Current system^69^, and off Peru^45^. Even though no such observations were made here, this could be a problem elsewhere in the world, if deep or submerged aquaculture cages were used. It is therefore relevant to predict and locate such potential deep-water hypoxia upwellings. However, this phenomenon is not at present a concern in Norwegian waters. This is because the oceanic oxygen conditions at intermediate depths in the Norwegian Sea are fundamentally different. Here, dense-water formation and associated ventilation in the Greenland Sea brings cold oxygen rich water downwards. This ventilated water in part spreads out in the Norwegian Basin forming a layer of relative oxygen maximum^70^. Thus, even during prevailing upwellings during spring, the resulting deeper water entering the shelf at depth would still be relatively high in oxygen.

### Method consideration for correcting oxygen data

In the study we used accurate oxygen data from the Torungen-Hirtshals section obtained by the Winkler method to justify the assumption of oxygen saturation at the surface in data sampled in other regions. There is a clear seasonal cycle in the oxygen saturation at the surface but limited to ± 5% of saturation. A similar seasonal cycle is expected along the Norwegian Coast but with a northward delay^71^. Since this delay is not accurately known and may vary from year to year, we have here chosen the simpler approach of assuming 100% oxygen saturation at the surface. Hence, the corrected oxygen data obtained by the optode are likely over-/underestimated during months with under/-oversaturation (Fig. 6). However, this error will be relatively small. Also, the relative rapid gas exchange of oxygen at the air-ocean interface supports the assumption of oxygen saturation in the surface water^42,57^. We therefore feel confident that this is a robust assumption and that we can use the oxygen data obtained with the optode to describe the seasonal development of oxygen variability on the Norwegian shelf.

## Data Availability

The datasets used in the current study are available from the corresponding author on reasonable request.
